# Prevalence of 845G>A *HFE* mutation in Slavic populations: an east-west linear gradient in South Slavs

**DOI:** 10.3325/cmj.2011.52.351

**Published:** 2011-06

**Authors:** Grażyna Adler, Jeremy S Clark, Beata Łoniewska, Andrzej Ciechanowicz

**Affiliations:** 1 Department of Medical Biology, Pomeranian Medical University, Szczecin, Poland; 2 Department of Clinical and Molecular Biochemistry, Pomeranian Medical University, Szczecin, Poland; 3 Department of Neonatal Diseases, Pomeranian Medical University, Szczecin, Poland; * Contributed equally to the study.

## Abstract

**Aim:**

To compare A allele frequencies of the 845G>A mutation of 10 Slavic populations in central, eastern, and southern Europe between each other and with other European populations.

**Methods:**

The 845G>A mutation from the DNA of 400 Polish neonates collected in 2005-2006 was analyzed by polymerase chain reaction-restriction fragment length polymorphism. The data were compared with reports from other countries.

**Results:**

We identified 381 GG homozygotes, 18 GA heterozygotes, and 1 AA homozygote. The 845A allele frequency was 2.5%, which makes the summary figure for Poland from this and previous studies 3.5%. The average prevalence for Poland and other West Slavic countries was 3.6%, similar to Russia (inhabited by the East Slavs, 3.5%). The average prevalence in South Slavic countries was 2.2%, gradually decreasing from 3.6% in Slovenia to 0% in Bulgaria, with a longitudinal linear gradient (adjusted R^2^ = 0.976, *P* < 0.001).

**Conclusions:**

The West and East Slavs, together with Finland, Estonia, Germany, Austria, Hungary, Slovenia, and Croatia, form a group with 845A allele frequencies between 3% and 4%. In the South Slavs, there is a gradual decline in the prevalence of 845A allele from northwest to southeast, with a surprisingly exact east-west linear gradient.

In 1996, two major *HFE* gene mutations (845G>A and 187C>G) responsible for an inherited form of hemochromatosis were identified (1). Hereditary hemochromatosis is a common autosomal recessive disorder characterized by increased iron absorption. It has significant clinical consequences such as liver cirrhosis, diabetes mellitus, arthropathy, cardiomyopathy, and endocrine dysfunction (2). A total of 60% to 96% of patients with hemochromatosis in Europe have the mutation 845G>A in exon 4. This causes cysteine to tyrosine substitution at position 282 (C282Y) of the polypeptide chain, resulting in destabilization of one of the bridging sulfide molecules disrupting HFE binding to β2-macroglobulin (1,3). The HFE polypeptide chain loses its ability to bind to transferrin receptor, and this results in a 200-300% increase in iron absorption from food. The severity of symptoms in homozygotes is variable and depends on the race, age, sex, and diet (2,4,5). Merryweather-Clarke et al (6) reported the highest prevalence of 845A *HFE* in northwestern Europe (5.2 to 10.1%), ie, Sweden, Norway, UK, and Ireland. In Finland, Hungary, Poland, Russia, Austria, Germany, Czech Republic, and Slovakia the prevalence was between 3.2 and 4%. In southern Europe (Greece, Romania, Italy, and Spain), the prevalence is very low (6-18) and in Turkey it is almost non-existent (7). According to more recent data, France (6.1%) can now be added to the northwestern group (19,20). As the major comparison of the prevalence between European countries by Merryweather-Clarke et al (6) included few data on Slavic populations, we further assessed the 845A *HFE* frequency in the Polish population and compared it with other Slavic populations and previously published results, as well as determined its distribution across the entire Europe.

## Materials and methods

The study sample comprised 400 consecutively born neonates (187 female and 312 male) delivered at the Neonatology Department, Pomeranian Medical University, Szczecin, Poland in 2005-2006. All neonates were of Polish origin, with Polish grandparents, and informed consent was obtained from all parents. The Ethical Committee of the Pomeranian Medical University approved the protocol of the study (BN- 001/57/05). Genomic DNA from neonates was extracted from 100 μL of umbilical cord blood using the QIAamp DNA Blood Mini Kit (QIAGEN, Hilden, Germany). For identification of the 845G>A *HFE* mutation, we used polymerase chain reaction (PCR)-restriction fragment length polymorphism. About 20 ng of genomic DNA was used with a PCR mixture (10 μL) containing 10 × buffer (pH 8.3, 1.5 mM MgCl_2_), 0.2 mM each of the deoxynucleoide triphosphates, 0.5 U Polymerase Taq (MBI Fermentas, Vilnius, Lithuania), and 4 pmol each of the forward and reverse primers. 5′- CCT CAT CCT TCC TCT TTC CT-3` was used as a forward primer and 5′- TCC TCA GGC ACT CCT CTC AA-3` as a reverse primer (TIB MOL BIOL, Poznan, Poland). PCRs were performed in a Mastercycler Gradient thermal cycler (Eppendorf, Hamburg, Germany), with the following temperature profiles: initial denaturation at 94°C for 5 minutes, 37 cycles of 20 seconds at 94°C, 40 seconds at 54°C, and 40 seconds at 72°C; with a final extension step at 72°C for 8 minutes. Amplification was followed by digestion of the 367 bp product using the RsaI restriction enzyme (5′-GT↓AC-3′) (MBI Fermentas) for 3.5 hours at 37°C. PCR digestion products were separated on 3% agarose gels, stained with ethidium bromide, and recorded using a DS-34 Polaroid Instant Camera (Polaroid, Dreieich, Germany) under UV light (Transilluminator 4000, Stratagene, La Jolla, CA, USA). The RsaI digestion yields fragments of 225 and 142 bp for G845 homozygotes; 225, 142, 113, and 29 bp for heterozygotes; or 225, 113, and 29 bp for 845A homozygotes. Genotypes of GA and AA patients were also confirmed by DNA sequencing (3100-Avant Genetic Analyzer, Applied Biosystems Hitachi, Foster City, CA, USA).

### Statistical analysis

Geographical coordinates (longitude) used in the analysis were derived from Google maps (Google Inc, Mountain View, CA, USA), except in cases of South Slavic countries, where additional sources were used for Serbia and Montenegro (*http://www.mapcrow.info/cgi-bin/cities_distance_airpt.cgi?city3=9173706%2C00&city4=135261%2C00*) and other South Slavic countries (*http://universimmedia.pagesperso-orange.fr/geo/loc.htm*). Graphical materials were developed using Designworks software (GSP Ltd, London, UK) and Microsoft Office (Microsoft, Redmond, WA, USA). Linear regression analysis was performed using STATISTICA, version 8.0 (StatSoft, Inc, Tulsa, OK, USA, *www.statsoft.com*), with the significance level set at *P* < 0.05.

## Results

The frequency of the 845A allele in 400 Polish neonates was 2.5%. The 845G>A genotype distribution was 381 GG homozygotes, 18 GA heterozygotes, and 1 AA homozygote. The 845G>A genotype distribution conformed to the expected Hardy-Weinberg equilibrium. The frequency of 845A *HFE* in 10 Slavic countries varied from 2.5% to 5.0% (Table 1).

**Table 1:** Allele frequencies for 845G>A *HFE* in Slavic countries

Population	Reference	Number of participants		Frequency of 845A allele (%)	
per study	sum per country	per study	per country*
West Slavs					
Poland	Present study	400 (neonates)	2788	*2.5*	3.5
	Raszeja-Wyszomirska J et al, 2008 (21)	1517 (healthy adults)		4.0	
	Moczulski DA et al, 2001 (22)	871 (healthy adults)		3.1	
Czech Republic	Cimburová M et al, 2005 (14)	481 (neonates)	620	3.4	3.8
	Zdárský E et al, 1998 (10)	139 (healthy adults)		5.0	
Slovakia	Gabrikova D et al, 2011 (23)	359 (general population)	359	4.0	4.0
South Slavs					
Slovenia	Cukajti M et al, 2007 (24)	1282 (blood donors)	1779	3.6	3.6
	Hruškovičová H et al, 2005 (25)	115 (healthy adults)		3.0	
	Zorc M et al, 2004 (26)	182 (adults)		4.1	
	Ristić S et al, 2003 (27)	200 (blood donors)		3.3	
Croatia	Starčević- Čizmarević N et al, 2006 (28)	350 (healthy adults)	550	3.4	3.4
	Ristić S et al, 2003 (27)	200 (healthy adults)		3.3	
Bosnia and Herzegovina	Terzić R et al, 2006 (29)	200 (blood donors)	400	2.0	2.2
	Hercegovac A et al, 2008 (30)	200 (healthy adults)		2.3	
Serbia and Montenegro	Šarić M et al, 2006 (31)	318 (healthy adults)	318	1.6	1.6
Macedonia	Arsov T et al, 2005 (32)	100 (healthy adults)	100	1.0	1.0
Bulgaria	Ivanova A et al, 1999 (33)	100 (healthy adults)	100	0.0	0.0
East Slavs					
Russia (European part)	Kondrashova V et al, 2006 (34)	260 (healthy women)	1100	3.3	3.5
	Pothekina E S et al, 2005 (35)	840 (blood donors)		3.5	
Belarus	no data				
Ukraine	no data				

*Weighted average.

**Figure 1:**
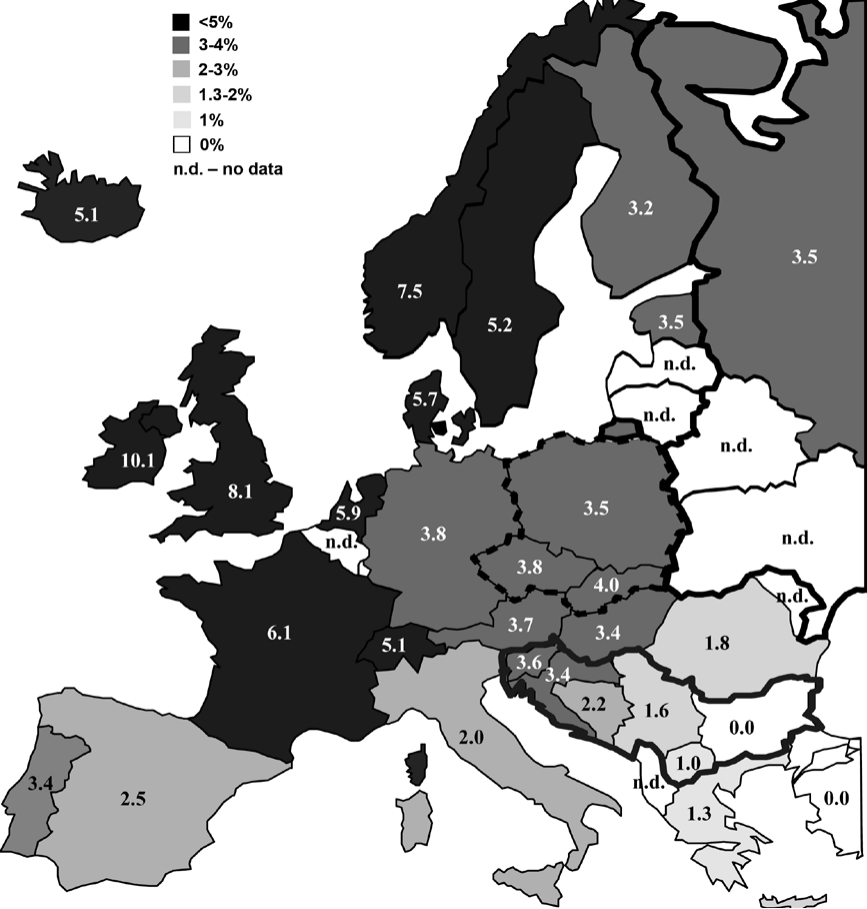
Allele frequencies for the 845G>A *HFE* mutation. Global averages for countries inhabited by the West Slavs – dashed line, South Slavs – lower solid thick line, and East Slavs – upper solid thick line (references as in Table 1). Global averages for other countries – Austria (36), Denmark (37), Estonia (38,39), Finland (7,40-42), France (19,20), Greece (7), Germany (7-9), Hungary (43), Iceland (7,44), both (45), Ireland (6,7,46-48), Italy (7,12,13,15,16,49-52), Netherlands (53,54), Norway (7,55,56), Portugal (57), Romania (18), Spain (7,11,17,58-61), Sweden (40), Switzerland (62), Turkey (7), UK (6,7,63-67). n.d. – no data.

The average prevalence of 845A allele in the countries inhabited by the West Slavs was 3.6% and varied from 3.5% in Poland (including our study) through 3.8% in the Czech Republic to 4.0% in Slovakia (10,14,21-23) (Figure 1). This places the West Slavs to a group with allele frequencies between 3% and 4%, together with East Slavic Russia (3.5%) and Finland, Estonia, Germany, Austria, Hungary, Slovenia, and Croatia.

**Figure 2:**
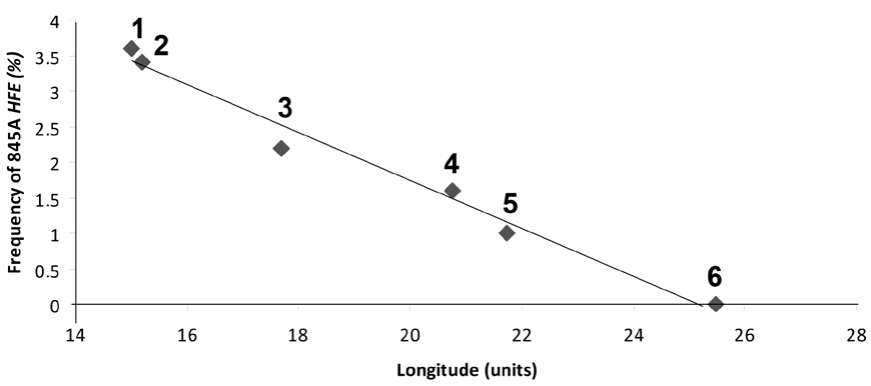
Linear trend in the frequency of 845A *HFE* with increase in longitude. 1 – Slovenia; 2 – Croatia; 3 – Bosnia and Herzegovina; 4 – Serbia and Montenegro; 5 – Macedonia; 6 – Bulgaria. Adjusted R^2^ = 0.976, *P* < 0.001

The average prevalence of the 845A allele in South Slavic countries was 2.2%. The highest prevalence was observed in Slovenia (3.6%) (24-26), after which a gradual northwest-southeast decrease was observed: 3.4% in Croatia, 2.2% in Bosnia and Herzegovina, 1.6% in Serbia and Montenegro, 1.0% in Macedonia, and 0% in Bulgaria (27-30,32,33). When allele frequency was plotted against geographical longitude, a surprisingly exact east-west linear gradient was obtained (adjusted R^2^ = 0.976, *P* < 0.001; Figure 2).

## Discussion

Our study showed that the distribution of 845A *HFE* among Slavic countries was bi-modal. The West and East Slavs had a similar prevalence as other central and eastern European populations, with values ranging between 3% and 4%. The South Slavs exhibited a linear decreasing west-to-east trend, with a prevalence varying from 3.6% to 0%.

Population migrations in Europe have led to the distribution of ethnic groups and cultures, and consequently to genetic mixing (68). Migrations, together with other factors, have also determined the prevalence of genetic diseases, such as hemochromatosis (68). Initially, the mutation 845G>A *HFE* was described in populations of northwestern European origin and has spread to territories inhabited by the Celts (68). On the other hand, the 845G>A mutation might have originated from the Germanic Iron Age population in southern Scandinavia and spread with the Vikings (37,68). In either case, this would result in lower 845A allele prevalence in the areas where their expansion was limited: central Europe, the Balkans, and Mediterranean countries.

Our study, together with the study by Merryweather-Clarke et al (6), suggests that a group of countries in central and east Europe have similar prevalence of the mutation. To our knowledge, this is the first comprehensive comparison of the 845A *HFE* mutation prevalence between all West and South Slavic populations.

Among the South Slavs, there was a linear gradual decrease in the prevalence of allele 845A *HFE*. This linearity suggests a possible stability and demic diffusion of the genes from the northwest into a block (the South Slavs) in which there are no conditions for the maintenance of a high frequency of the mutation (either because of genetic background or environmental reasons). An alternative explanation is that there is a gradient of conditions to which South Slavic populations have been exposed, forming a gradient of positive selection and heterozygous advantage. A third possibility, perhaps supported by Y-chromosome haplotypes, is that the medieval expansion of the Slavs (beginning during the 5th and 6th century) resulted in a gradient of ancestors carrying this allele (69).

It would be interesting to see if such a gradient is found with mutations in other genes and to compare this with genetic distance data both among the Southern Slavs and the surrounding populations. Additionally, a spatial frequency distribution map constructed using intra-country regions would be of benefit – especially as a gradient similar to the one described here (but across one country) has been found in Portugal (57). It would also be interesting to fill in the gaps for the East Slavic populations, including those in Belarus, Ukraine, and the West Slavic group in Germany (the Sorbs).
